# 170. Comparison of Traditional Stool Culture and Targeted Testing Using Multiplex PCR to Diagnose Infectious Diarrhea at the Atlanta VA Medical Center (AVAMC): A Diagnostic Stewardship Initiative.

**DOI:** 10.1093/ofid/ofac492.248

**Published:** 2022-12-15

**Authors:** Alexander H Molinari, Lauren H Epstein, Nicole Darius, Tiffany Goolsby, Andrew S Webster

**Affiliations:** Emory University, Atlanta, Georgia; Emory University School of Medicine, Atlanta, Georgia; Atlanta VAMC, Atlanta, Georgia; Atlanta VAMC, Atlanta, Georgia; Atlanta VA Medical Center, Decatur, Georgia

## Abstract

**Background:**

Multiplex PCR panels have become common for diagnosing infectious diarrhea; however, indiscriminate use of multiplex PCR panels may identify common pathogens that do not require treatment.

To optimize laboratory workflow and improve diagnostic stewardship, we developed a targeted algorithm to optimize multiplex PCR testing at the AVAMC. We retroactively applied the targeted algorithm to historical stool cultures to assess the potential impact on diagnosis, inappropriate test ordering, and cost.

**Figure 1:**
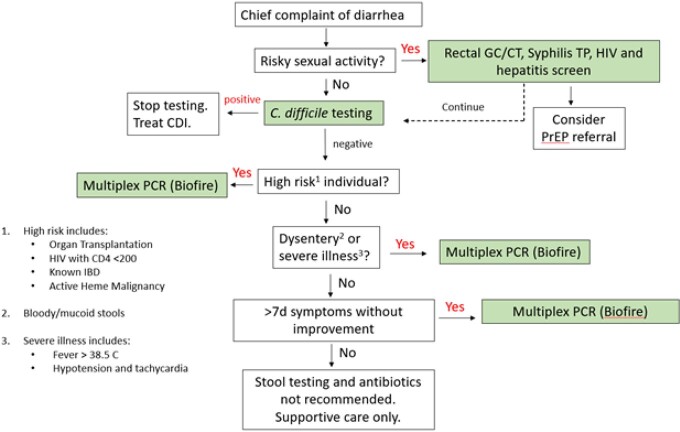
Targeted Algorithm for Testing Infectious Diarrhea

**Methods:**

In March 2022, multiplex PCR testing (Biofire) was initiated at AVAMC; we developed a targeted algorithm to inform testing of patients at highest risk from infectious bacterial diarrhea (Figure 1). We retrospectively reviewed all clinical information among patients with bacterial stool testing, which included cultures and antigen tests, obtained from January 1, 2019 to December 31, 2021. In our analysis, we included all patients with a positive stool culture and a random sample of patients with a negative stool culture. We applied the targeted algorithm to both retrospective cohorts in order to evaluate potentially missed cases, outcomes, and overall differences in costs and labor between traditional stool cultures and targeted multiplex PCR testing.

**Results:**

We identified 1027 patients with bacterial stool testing. Among 27 patients with a positive stool test, the targeted algorithm missed testing among 6 (22%) patients with mild, self-limited disease that resolved within 7 days. Among 100 patients with negative stool cultures, the targeted algorithm excluded testing among 75 (75%) patients. Among the remaining 25 (25%) negative patients who would have been tested using the targeted algorithm, 14 (56%) were high-risk and 16 (64%) had severe illness. Eliminating unnecessary stool testing using a targeted algorithm would have saved an estimated $50,000 and 750 laboratory labor hours.

**Conclusion:**

At our facility, the implementation of algorithm-guided infectious diarrhea testing by multiplex PCR is estimated to reduce laboratory time and materials costs while eliminating 75% of unnecessary testing. Algorithm-guided testing would capture the majority of bacterial diarrhea at our institution but could lead to missed diagnoses in lower-risk populations.

**Disclosures:**

**All Authors**: No reported disclosures.

